# A Systematic Review of Evidence-Based Cognitive and/or Behavioural Interventions Targeting Mental Health in LGBTQ+ Populations

**DOI:** 10.32872/cpe.11323

**Published:** 2024-09-30

**Authors:** Carina Tudor-Sfetea, Raluca Topciu

**Affiliations:** 1 CEDAR (Clinical Education Development and Research), University of Exeter, Exeter, United Kingdom; Friedrich-Alexander-Universität Erlangen-Nürnberg, Erlangen, Germany

**Keywords:** LGBTQ+, systematic review, mental health, cognitive behavioural interventions

## Abstract

**Background:**

Despite a minority stress-related higher risk to develop mental health difficulties, and problematic access to and treatment from healthcare providers, research into LGBTQ+ mental health support is limited. The aims of this systematic review were to explore evidence-based cognitive and/or behavioural interventions and adaptations targeting mental health in LGBTQ+ populations, before providing recommendations for future clinical and research directions.

**Method:**

Six databases were searched in February-March 2022 and risk of bias evaluated using the Cochrane RoB 2/ROBINS-I tools. A narrative synthesis following the PICOS framework and the review questions was used to examine the results.

**Results:**

Sixteen studies met inclusion criteria, including various interventions and adaptations, mental health difficulties, and other emotion- and minority stress-related processes/constructs. Risk of bias was judged as high, and critical/serious, respectively, in all studies. Outcomes included improvements in symptoms of depression (most statistically/clinically significant effects/large effect sizes), and anxiety, emotion regulation, and internalised homophobia in the pre-post studies.

**Conclusion:**

Cognitive/behavioural interventions and adaptations for LGBTQ+ populations feature a range of therapeutic modalities and levels of adaptation, with largely positive effects, in the context of limited and heterogenous literature and risk of bias concerns, as well as limitations related to publication bias and inclusion criteria of the current work. Suggestions for future clinical and research directions include a focus on generic therapeutic competencies and metacompetencies, and affirmative, potentially more holistic approaches, as well as more consistency in methodology, more focus on underserved LGBTQ+ populations and intersectionality, and more detailed investigations into mechanisms of change.

## Highlights

- Research findings on evidence-based mental health support for LGBTQ+ individuals are positive, but the literature is limited, heterogenous, and there are risk-of-bias concerns.
- More work is needed around affirmative approaches, consistency in methodology, mechanisms of change, and underserved LGBTQ+ populations and intersectionality.

## Rationale

LGBTQ+ individuals (who identify as lesbian, gay, bisexual, transgender, queer, or with any other non-heterosexual and/or non-cisgender identity) experience a disproportionately higher rate of mental health difficulties compared to heterosexual and/or cisgender individuals (Pinna et al., 2022; Plöderl & Tremblay, 2015). This disparity has been attributed at least partly to stigma-related stressors, with perhaps the most important framework addressing this being the Minority Stress Theory (Brooks, 1981; Meyer, 1995, 2003; reviewed in Hoy-Ellis, 2023; Tan et al., 2020) alongside its extensions, particularly Hendricks and Testa’s (2012) work exploring gender identity stressors.

Herein, distal (external, objective) factors – victimisation, prejudice, and discrimination, and the likely resulting proximal (internal, subjective) factors – concealment of one’s identity, prejudice- and rejection-related anxiety and expectations, and internalised homo- and transphobia, are thought to contribute to a set of differences in cognitions, emotions, and behaviours which drive and maintain mental health disparities transdiagnostically (Meyer, 2003; Nicholson et al., 2022; Pachankis, 2015). The effects of these factors have been widely documented (Gnan et al., 2019; Testa et al., 2017). A complicating, yet crucial, consideration, is that of the intersection of various sexual identities with other racial, ethnic, social, and gender identities, with individuals with multidimensional minority status facing unique challenges (Balsam et al., 2011; Dale & Safren, 2019).

Various mechanisms have been proposed in the context of minority stress. These include: alterations in emotion regulation, social/interpersonal dynamics (e.g., isolation), and cognitive processes (e.g., negative self-schemas; Hatzenbuehler, 2009); disruptions of negative valence systems (avoidance, hypervigilance, loss), positive valence systems (approach motivation, reward learning – associated with impulsivity/addictive behaviours), social functioning (e.g., disrupted attachment, low agency, poor social communication; Pachankis, 2015), and anticipatory emotions/behaviours as well as cognitions around the expectation of rejection (Feinstein, 2020). Biological mechanisms (Flentje et al., 2020) and neuroimaging/neural correlates (Nicholson et al., 2022) have also been documented.

Despite these significant vulnerabilities, access to and treatment for mental health seems to be problematic for LGTBQ+ populations (e.g., McCann & Sharek, 2014; Steele et al., 2017). While some limited research has documented poorer psychological treatment outcomes for some LGBTQ+ populations (Beard et al., 2017; Rimes et al., 2019), there is generally a paucity of literature (e.g., data pertaining to sexual orientation and gender identity is often omitted in research on psychological interventions for mental health – Heck et al., 2017). This speaks to the need for tailored mental health interventions for this population, and crucially, thorough research into their effectiveness.

Others have reviewed interventions targeting mental health and/or health behaviour in various LGBTQ+ sub-populations. In their systematic review and meta-analysis, Pantalone et al. (2020) focused on behavioural interventions targeting psychosocial syndemics and HIV-related health behaviours for sexual minority men, reporting significant improvements with small effect sizes in mental health, while a systematic review by Melendez-Torres and Bonell (2014) found improvements related to sexual risk behaviour following a CBT (Cognitive Behavioural Therapy) intervention in substance-using men who have sex with men, although the evidence was evaluated to be of moderate quality. Focusing on LGBTQ+ youth mental health, Hobaica et al. (2018) found support for the effectiveness of a range of intervention modes, including in-person, computerised, online, as well as individual and group. Sheinfil et al. (2019) investigated adapted psychotherapeutic interventions for depression, while Van Der Pol-Harney and McAloon (2019) found CBT to be an effective therapeutic framework. Bochicchio et al. (2022) also reported preliminary evidence for effectiveness of a variety of psychotherapeutic interventions.

This work has, however, either mainly focused on health behaviour rather than mental health, therefore not including details around intervention components, outcome measures, and their relationship to minority stress (Pantalone et al., 2020); on specific genders or populations known to present with unique challenges (sexual minority men including those HIV-positive or at risk – Pantalone et al., 2020; substance-using sexual minority men – Melendez-Torres & Bonell, 2014; young people – Bochicchio et al., 2022; Hobaica et al., 2018; Van Der Pol-Harney & McAloon, 2019), therefore making generalisations limited; or on particular diagnoses rather than more widely/transdiagnostically which would be more in line with minority stress factors and mechanisms (Sheinfil et al., 2019).

Few, if any reviews have adopted a wider/more general lens on LGBTQ+ populations of any age, focusing on psychotherapies for mental health and their adaptations, their components, their outcomes, and their relationship to transdiagnostic minority stressors; this review aims to bridge this gap. As CBT has a rich evidence base for several mental health difficulties (Hofmann et al., 2012), and importantly, offers a framework by which to understand and explore minority stressors (i.e, relationships among cognition – e.g., negative self-schemas, emotion – e.g., emotion regulation, anxiety, shame, and behaviour – e.g., isolation, avoidance), the review will focus on this psychotherapeutic model.

## Objectives

The aim of this systematic review is to explore the landscape of the scientific literature on evidence-based cognitive and/or behavioural interventions and adaptations targeting mental health in LGBTQ+ populations, by answering the following questions:

1. What evidence-based cognitive and/or behavioural interventions for LGBTQ+ populations exist, and what, if any, specific adaptations do they involve?
2. What are the outcomes of evidence-based cognitive and/or behavioural interventions and adaptations targeting mental health in LGBTQ+ populations?
3. What recommendations could be made in terms of such adaptations in clinical practice?

## Method

### Guidelines and Registration

This systematic review was carried out in accordance with the updated PRISMA guidelines (Page et al., 2021), and registered on PROSPERO (International prospective register of systematic reviews) in April 2022 (CRD42022243466) – please see Tudor-Sfetea and Topciu, 2024S, Appendix A for more information regarding deviations from this preregistration. No ethics approval was required due to the nature of the work.

### Eligibility Criteria

Studies had to be published or in press in peer-reviewed journals, in English; no time limits for publication were enforced. Pre-prints were considered, while other grey literature was excluded. The studies also had to fulfil the criteria outlined in Table 1, following the PICOS framework (Population, Intervention, Comparison, Outcomes, Study designs, Higgins et al., 2023). Please see more details on these decisions in the Discussion section, and in Tudor-Sfetea & Topciu, 2024S, Appendix B.

**Table 1:** Inclusion and Exclusion Criteria According to the PICOS Framework

Inclusion	Exclusion
Population	
LGBTQ+ individuals or individuals reporting distress over minority stress-related issues, of any age, sex, gender, sexual orientation, race, and ethnicity; including people identifying as gay, lesbian, bisexual, pansexual, demisexual, asexual, queer, transgender, genderqueer, genderfluid, non-binary	Studies with HIV-positive participants where no separate results for participants with negative or unclear HIV status were reported, as well as studies with people with substance dependence as a main presenting problem
Interventions	
Evidence-based individual and group-based cognitive behavioural interventions; including Cognitive Behavioural Therapy (CBT), behaviour-based interventions such as exposure or exposure and response prevention (ERP), as well as third-wave CBT interventions, including Acceptance and Commitment Therapy (ACT), Mindfulness-Based Interventions such as Mindfulness-Based Stress Reduction (MBSR) and Mindfulness-Based Cognitive Therapy (MBCT), Dialectical Behaviour Therapy (DBT), Behavioural Activation (BA), or Compassion Focused Therapy (CFT); delivered in any settings, including out- and inpatient settings, charity organisations, educational settings, or any other community or home settings; and via any medium, including in person, videoconference, telephone, live-chat	Interventions with only a minimal cognitive or behavioural component, and couple-specific interventions; self-help interventions with no direct therapist involvement
Comparison	
Active control (i.e., other interventions for mental health; treatment-as-usual), inactive control (i.e., waitlist), or no control group	
Outcomes	
Outcomes in the domain of common mental health difficulties; including studies with outcomes related to, e.g., symptoms of depression, anxiety and any anxiety disorders, obsessive-compulsive disorder (OCD) and body dysmorphic disorder (BDD), health anxiety, post-traumatic stress disorder (PTSD), and minority stress, as well as psychological flexibility and quality of life/subjective wellbeing, assessed via validated questionnaires	Studies with outcomes related solely to sex-related health behaviour, as well as drug use
Study designs	
Quantitative studies or the quantitative aspects of mixed-method studies; including randomised controlled trials (RCTs), controlled/experimental studies such as controlled trials, open trials/studies/pilots, pilot trials/studies, case-control studies, effectiveness studies without a control group (e.g., pre-post effect size), feasibility or acceptability studies	Qualitative studies, as well as published study protocols and reviews

### Information Sources

Eligible studies were sourced from: Embase, MEDLINE, PsycINFO, PsychExtra, Web of Science, Cochrane Library (advanced search), via searches between 19.02.2022 and 10.03.2022.

### Search Strategy

Search terms based on the PICOS framework were used to determine MeSH (Medical Subject Heading) terms where applicable, and perform searches using these as well as keyword searches, combined with Boolean logic, OR/AND – a table of the search terms, and a link to the full search strategy/history are available in Tudor-Sfetea and Topciu, 2024S, Appendix C.

Results were exported into RIS and Microsoft Excel files, before being imported into Covidence (Veritas Health Innovation, 2022), a screening and data extraction tool recommended for Cochrane authors.

### Selection Process

Duplicates were automatically removed in Covidence. Reference titles and abstracts were then screened by the first author and categorised as “Yes”, “No”, “Maybe”, before reviewing the full texts of the “Yes” and “Maybe” references. A second reviewer followed the same process for a randomly-selected subset, at both stages (approximately 20%; *n* = 51, *n* = 5 respectively). Disagreements (*n* = 5 at title and abstract screening stage, none at full text review stage) were resolved by discussion and revisiting/clarification of criteria, with consensus reached throughout.

### Data Collection Process

Data were extracted using customised forms on Covidence, based on the Cochrane Data collection forms for intervention reviews. The forms were piloted on one randomly selected study, and further refined. A subset (12.5%, *n* = 2) of the extracted data were checked for accuracy by the second reviewer; no disagreements occurred. A link to a more extensive, raw data table is available in Tudor-Sfetea and Topciu, 2024S, Appendix D.

### Study Risk of Bias Assessment

All included studies were assessed for risk of bias. There seems to be no agreed standard to evaluate the quality of psychotherapy outcome research; instead, a heterogeneity of tools are available, with the Cochrane tools or adapted versions thereof more common (Munder & Barth, 2018). Therefore, the Cochrane risk-of-bias tool for randomised trials (RoB 2) (Sterne et al., 2019) for randomised studies, and the Risk of Bias In Non-Randomised Studies - of Interventions (ROBINS-I) tool (Sterne et al., 2016) for non-randomised studies were used, consistent with PRISMA guidelines (Page et al., 2021), and to align to the majority of previous research, encouraging consistency and reproducibility.

The tool domains were evaluated categorically as *Low*, *High*, or *Some concerns* (RoB2), or *Low*, *Moderate*, *Serious*, *Critical*, or *No information* (ROBINS-I), in line with the signalling questions and guidance (Sterne et al., 2019; Sterne et al., 2016, respectively). Customised Quality Assessment Templates on Covidence were used. A subset (12.5%, *n* = 2) of the studies were also evaluated by the second reviewer; no disagreements occurred.

### Synthesis Methods

Due to the limited number of included studies and the heterogeneity of results in terms of study designs and outcomes, following scoping/initial searches, the data were deemed not appropriate for quantitative synthesis. Therefore, a narrative synthesis considering the “Synthesis without meta-analysis" (SWiM) guidelines (Campbell et al., 2020), as well as tables and figures, were used to summarise and explain the characteristics of the included studies.

## Results

### Study Selection

**Figure 1:**
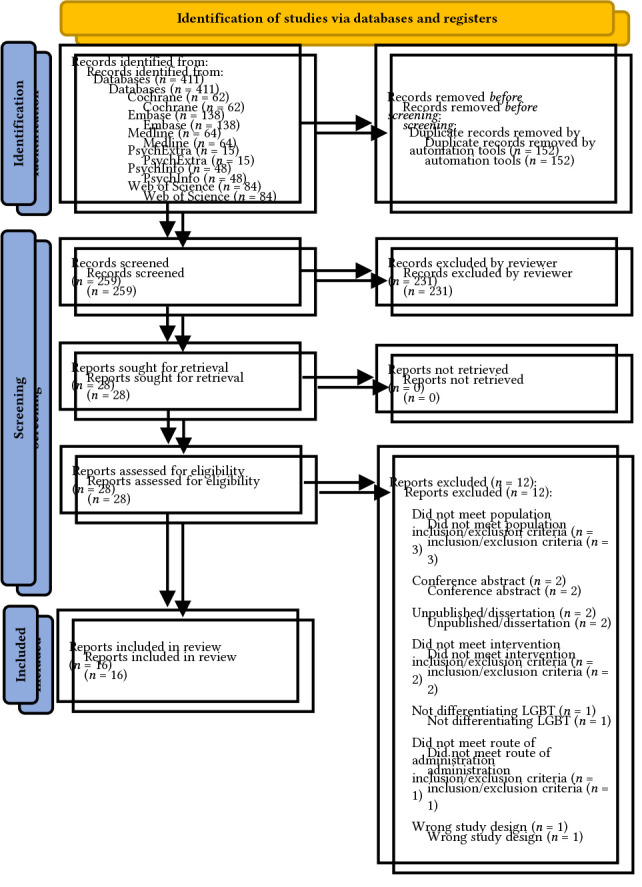
PRISMA Flow Diagram Outlining the Process of Study Selection

A total of 411 records were identified and imported into Covidence, with 152 records automatically identified as duplicates and removed. Of the remaining 259 records, 231 were excluded following title and abstract screening, resulting in 28 records eligible for full text review. Twelve of these were then excluded as they did not meet the review criteria; see Tudor-Sfetea and Topciu, 2024S, Appendix E for a detailed overview. Therefore, 16 records were included. The PRISMA flow diagram in Figure 1 outlines this process.

### Study Characteristics

Table 2 summarises study and sample characteristics. Studies have been numbered for clarity (chronologically, starting with the oldest, grouped by RCTs, then non-RCTs), and will be referred to by their allocated numbers from now on. Please see Tudor-Sfetea and Topciu, 2024S, Appendix F for a narrative summary of study and sample characteristics.

**Table 2:** Study and Sample Characteristics

Study; year(s) of data collection; country/countries of data collection	Study designs; Comparisons	Timepoints of outcome collection	Sample size	Participants				
				Age - *M* (*SD*) / Breakdown	Gender (% or *N*)	Sexual orientation (% or *N*)	Ethnicity (% or *N*)	Mental health
1 - Pachankis et al. (2015); 2013-2014; USA	Randomised controlled trial (crossover - participants were randomised (stratified according to race/ethnicity and anxiety/depression) to either immediate treatment - received treatment between baseline and 3-month assessment -, or waitlist - received treatment between 3-month and 6-month assessment -); Inactive control (waitlist)	Immediate condition: pre-treatment, post-treatment, 3-month follow-up; Waitlist condition: 3-month pre-treatment, pre-treatment, post-treatment	63 (54 completed at least one session)	Immediate condition: 26.19 (4.26); Waitlist condition: 25.69 (4.28)	Male - inclusion criterion;	Gay/queer: 31; 27 Bisexual: 1; 4	Immediate condition; Waitlist condition: American Indian/Alaskan Native: 0; 1 Asian: 0; 3 Black/African American: 6; 4 Pacific Islander: 1; 1 White: 16; 17 Other/mixed: 9; 5 Hispanic/Latino: Yes - 12; 11; No - 20; 20	Immediate pre-treatment mean scores (*SD*) Depression: CESD - immediate condition: 27.69 (1.83), waitlist condition: 23.19 (2.14) - above cut-off 16 ODSIS - immediate condition: 8.16 (0.76), waitlist condition: 7.08 (0.88) - just above /slightly below cut-off 8 Anxiety: OASIS - immediate condition: 8.03 (0.66), waitlist condition: 6.89 (0.78) - just above/slightly below cut-off 8
2 - Millar, Wang, & Pachankis (2016); 2013-2014; USA	As above	As above	63 enrolled, 54 completed both pre- and post-treatment assessments	*M* = 26.1 (*SD* = 4.0)	Male - inclusion criterion	Gay/queer (49), Bisexual (4)	American Indian or Alaskan Native (1), Asian (1), Black / African American (7), Pacific Islander (2), White (30), Other/mixed (13) Hispanic / Latino - Yes (22), No (32)	Pre-treatment mean scores (*SD*) Depression: ODSIS - 7.46 (4.30) - below cut-off 8 Anxiety: OASIS - 7.50 (3.76) - below cut-off 8
3 – O’Cleirigh et al. (2019); 2007-2011; USA	Randomised controlled trial; active control (VCT-only)	Baseline, end of the treatment period (approximately 3-months after randomization), and 6- and 9-month follow-up	43	*M* = 39.19 (*SD* = 11.07)	Male - inclusion criterion	Gay (27), Bisexual (12), Unsure (4)	Caucasian (27), African American (11), Hispanic/Latino (3), Other (2)	Baseline mean scores (*SD*) PTSD: Davidson Trauma Scale Control - 37.20 (25.29) - below cut-off 40 Treatment - 47.09 (21.27) - above cut-off 40 Report also states 32.6% of participants met diagnostic criteria for PTSD
4 - Pachankis et al. (2020); 2018-2019; USA	As Pachankis et al. (2015) and Millar, Wang, & Pachankis (2016)	As Pachankis et al. (2015) and Millar, Wang, & Pachankis (2016)	60 enrolled, 58 completed at least one session	*M* = 25.58 (*SD* = 3.26)	Women - inclusion criterion; cisgender (56.7%)	Queer (55%)	White (58.3%), racial or ethnic minorities (41.7%)	Immediate pre-treatment mean scores (*SD*) Depression: CESD - immediate condition: 29.70 (1.84), waitlist condition: 26.86 (1.91) - above cut-off 16 ODSIS - immediate condition: 6.30 (0.83), waitlist condition: 7.69 (0.73) - below cut-off 8 Anxiety: OASIS - immediate condition: 8.80 (0.64), waitlist condition: 8.03 (0.46) - just above cut-off 8
5 - Maguen, Shipherd, & Harris (2005); unclear years of data collection; USA	Pre-post	Pre, post	6	*M* = 47 (*SD* = 9.16), range 32-59	Female (MtF)	N/A	N/A	Depression: 67% (4) scored above clinical threshold for BDI Anxiety: 67% (4) scored above clinical threshold for STAI
6 - Yadavaia & Hayes (2012); unclear years of data collection; USA	Concurrent, multiple-baseline, across-participants design (several coordinated simple phase changes, in which treatment begins for specific participants at different points in real time and after baseline periods of differing lengths); Pre-post	Pre, post, 4-week and 12-week follow-up	6 enrolled, 5 completed	Age 21-24 (3 participants), age >30 (1 participant), age 56 (1 participant)	Male (3), Female (2)	Gay (2), Lesbian (1), Questioning (1) - one ppt's data not reported due to preferences	Asian/African American/Caucasian (1), African American/Caucasian (1), Caucasian (1), Native American (1) - one ppt's data not reported due to preferences	Mean scores (*SD*) Depression: DASS-D - 14.4 (8.2) - indicating moderate depression Anxiety: DASS-A - 5.2 (3.9) - indicating normal anxiety
7 - Craig & Austin (2016); 2014; unclear countries of data collection – likely Canada, possibly USA	Open pilot, pre-post	Baseline (<4 weeks before start of intervention), post, 3-month follow-up	30	*M* = 16.8, range 15-18	Female (54%), gender independent/non-binary (21%), male (18%), trans (10%), and/or two-spirit (8%)	Pansexual (29%), lesbian (25%), queer (21%), bisexual (18%), unsure/questioning (11%), gay (11%), and/or polysexual (2%)	White European (64%), Black/African/Caribbean (25%), East/South/Southeast Asian (24%), Indigenous/First Nations (18%), and/or Latino/a (7%)	Mean scores (*SD*) Depression: BDI-II - 25.95 (14.51) - indicating moderate depression
8 - Austin, Craig, & D’Souza (2018); 2014; Canada	Pre-post	Pre, post, 3-month follow-up	8	Age 16 (1 participant), age 17 (1 participant), age 18 (6 participants)	Nonbinary (6), Queer (5), Female (2), Transgender (2), Male (1), Two-spirit (1), Gender independent (1), Other-figuring things out (1)	Queer (5), Pansexual (2), Questioning (2), Asexual (1)	White (Canadian, European) (5), Mixed (2), Asian (1), Black (African, Canadian, Caribbean) (1), Indigenous, First Nations, Inuit, Metis (1), Latin American (1)	Mean scores (*SD*) Depression: BDI-II - 37.50 (12.29) - indicating severe depression
9 - Jabson Tree & Patterson (2019); unclear years of data collection; USA	Pre-post	Pre, post, 12-week follow-up	24 enrolled, 17 completed	N/A	Female (11), Male (6)	Bisexual (1), Mostly lesbian/gay/homosexual (2), Only lesbian/gay/homosexual (12), Other (2)	N/A	N/A - no measures related to mental health disorders
10 - Cohen et al. (2021); unclear years of data collection; USA	Case series; Pre-post	Pre, post	7; 6 completed treatment	N/A	N/A	Sexual minority - unclear breakdown	N/A	Depression: 67% (4) scored above clinical threshold for PHQ-9 Anxiety: 50% (3) scored above clinical threshold for OASIS
11 - Hart et al. (2020); unclear years of data collection; unclear countries of data collection – Canada or USA	Pre-post pilot	Baseline, post, 3-, and 6-month follow-up	29 starters, 21 completers	*M* = 32.81 (*SD* = 8.95)	Male - inclusion criterion	Gay (18), Bisexual (3)	White (12), Black (2), East/Southeast Asian (0), Middle Eastern/North African (2), Latin American/Hispanic (2), Mixed Race (3)	Mean scores (*SD*) Social Anxiety: Liebowitz Social Anxiety Scale - 62.86 (22.76) - indicating moderate social anxiety SIAS - 47.38 (12.31) - above cut-off 34/36 SPS - 33.57 (18.81) - above cut-off 24 BFNE-S - 30.28 (7.11) - above cut-off 25 Report also states 95% of participants meeting diagnostic criteria for social anxiety Depression: CESD - 25.81 (12.76) - above cut-off 16 Report also states 24% of participants meeting diagnostic criteria for major depressive disorder, current episode
12 - Bluth et al. (2023); 2020-2021 (presumed due to mention of Covid-19 pandemic and 2021 year of publication); unclear countries of data collection – USA or Canada	Pre-post	Pre, post, 3-month follow-up	41	*M* = 14.5 (*SD* = 1.49)	Transgender M-F (9), Transgender F-M (18), Non-binary (12), Genderfluid (3), Questioning (2), Agender (1)	N/A	White (33), Black/African American (4), Asian (1), Hispanic/Latino/a (5), Other: Mixed (1)	Mean scores (*SD*) pre-intervention Anxiety: STAI - 50.77 (13.58) - above cut-off 40 Depression: PHQ-9 - 15.12 (6.77) - above cut-off 10
13 - Craig et al. (2021); 2020; unclear countries of data collection, likely Canada or USA	Non-randomised experimental study; inactive control	Pre, post	46 completers	*M* = 21.17 (*SD* = 4.52)	Non-binary (17), Transgender (14), Cis woman (8), Queer (3), Agender (2), Cis man (1), Two-spirit (0), Other (1)	Queer (12), Lesbian (10), Bisexual (6), Gay (6), Pansexual (6), Asexual (3), Questioning (2), Demi (1), Other (0)	White (35), Asian (5), Black (4), Middle Eastern (2), Indigenous (1), Latinx (0), Multi-ethnic/racial (5), Other (6)	Mean scores (*SD*) Depression: BDI-II - control: 19.48 (10.67), intervention: 19.30 (11.15) - indicating mild-moderate depression
14 - Pan et al. (2021); unclear years of data collection; China	Pre-post	Baseline, 1-month follow-up	8	Age 16-20 (2 participants), age 21-30 (3 participants), age >30 (3 participants)	Male - inclusion criterion	Gay (6), Bisexual (2)	Asian/Chinese (as per article title)	Baseline mean scores (*SD*) Depression: PHQ-9 - 10.43 (3.46) - above cut-off 10 Anxiety: GAD -7 - 7.43 (2.57) - below cut-off 8
15 - Jackson et al. (2022); 2018-2019; USA	Pre-post	Baseline, 3-month follow-up	21 starters, 17 completed the 3-month follow-up	Age 18-23 (4 participants), age 24-29 (11 participants), age 30-35 (6 participants)	Male - inclusion criterion; Cisgender man (20), Transgender man (1)	Gay (16), Bisexual (3), Queer (2)	Latino/Latinx (Hispanic) (7), White (Hispanic) (5), Black (Hispanic) 5, Black (non-Hispanic) (4)	Baseline mean scores (*SD*) Depression: CEDS - 22.10 (11.89) - above cut-off 16 ODSIS - 11 (4.79) - above cut-off 8 Anxiety: OASIS - 12.05 (3.54) - above cut-off 8
16 - Poon et al. (2022); unclear years of data collection; USA	Pre-post; LGBQ-non-LGBQ	Pre, post	39	*M* = 15.21 (*SD* = 1.65)	Female (86.8%)	LGBQ (16), Heterosexual (23)	Non-Hispanic White (71.1%), Hispanic (22.9%), bi- or multiracial (13.1%), Asian, African-American, or other (7.9%)	Pretreatment mean scores (*SD*) Depression: BDI-II - 28.64 (14.61) - indicating moderate-severe depression

### Interventions, Adaptations, and Results

Table 3 summarises the interventions, adaptations, and results of each of the included studies.

**Table 3:** Interventions, Adaptations, and Results

Study	Interventions	Any LGBTQ+-specific adaptations	Relevant outcomes (complete names and references in Tudor-Sfetea & Topciu, 2024S, Appendix I)	Relevant analyses; Number of participants included therein	Relevant results summary
1 - Pachankis et al. (2015)	CBT: ESTEEM intervention - 10 individually-delivered sessions, based on Barlow et al.’s Unified Protocol for the Transdiagnostic Treatment of Emotional Disorders	Yes - focus on the impact of minority stress on mental health, interpersonal functioning, unhelpful behaviours; aim of improving minority stress coping through emotion regulation, cognitive restructuring, assertiveness training	Center for Epidemiological Studies Depression Scale (CESD); Overall Depression Severity & Impairment Scale (ODSIS); Overall Anxiety Severity & Impairment Scale (OASIS); Measure of Gay-Related Stress (MOGS); Gay-related Rejection Sensitivity Scale (GRS); Internalized Homophobia Scale (IHS); Sexual Orientation Concealment Scale (SOCS); Ruminative Responses Scale (RRS); Difficulties of Emotion Regulation Scale (DERS); Rathus Assertiveness Schedule (RAS)	Linear mixed models with maximum likelihood estimation 1) Condition comparison 2) Generalized linear mixed models predicting the odds of meeting clinical cut-offs on CESD, ODSIS, OASIS 3) Pooled data (pre-treatment measures from the baseline assessment for the immediate participants and the three-month assessment for the waitlist participants, and post-treatment measures from the three-month assessment for the immediate participants and the six-month assessment for the waitlist participants) - change comparison across all participants from immediate pre-treatment to post-treatment 4) Follow-up assessment; 63 - intent-to-treat approach	1) Significant improvements in depressive symptoms (on ODSIS, not CESD), marginally significant improvements in anxiety (OASIS) in immediate vs waitlist condition (medium-large effects sizes), no significant condition - time interaction effects for cognitive, affective, and behavioural minority stress processes or for universal processes (small effect sizes) 2) Stronger decreases in the proportion of immediate versus waitlist participants who continued to exceed the cut-off at three months (on CESD, not ODSIS or OASIS) 3) Significant reductions in all primary outcomes, significant (apart from SOCS) reductions in all minority stress processes and universal processes from immediate pre-treatment to post-treatment (large effect sizes) 4) Treatment effects generally maintained at follow-up, few significant differences between post-treatment and follow-up, rumination scores continuing to significantly decrease from post-treatment
2 - Millar, Wang, & Pachankis (2016)	CBT: ESTEEM intervention	Yes - described above	Sexual Orientation Implicit Association Test; Internalized Homophobia Scale (IHS); Overall Depression Severity & Impairment Scale (ODSIS); Overall Anxiety Severity & Impairment Scale (OASIS)	Linear mixed models with maximum likelihood estimation, pooled data as above, two separate models - with implicit IH and explicit IH, and their respective interactions with time; 54 (who completed pre- and post-treatment assessments)	Depression and anxiety showed significant reductions; Participants higher in implicit IH at baseline showed nearly three times greater reductions than those lower in implicit IH on depression and anxiety; At post-treatment, those higher in implicit IH showed reductions on depression and anxiety roughly equivalent to one standard deviation
3 - O’Cleirigh et al. (2019)	CBT: 10-session integrated CBT for Trauma and Self-Care (CBT-TSC) intervention with HIV voluntary counseling and testing (VCT) or VCT alone (VCT-only)	Yes - participants in both conditions received HIV/STI voluntary counseling and testing (VCT) at baseline	Mini-International Neuropsychiatric Interview (MINI) - to assess symptoms and a diagnosis of PTSD Davidson PTSD Scale	HLM (Hierarchical Linear Modeling) 43	Davidson Trauma Scale Immediately post-treatment: - Significantly greater reductions in posttraumatic symptom severity for the CBT-TSC condition for the Total Score and the Avoidance subscale - Trend for a difference between the conditions for the Intrusions subscale Follow-up: - Trend for a statistically significant difference between the randomization conditions on the Total Score - Significant reductions in trauma symptom severity for the Avoidance subscale - Trend for a meaningful difference between the conditions for the Intrusions subscale
4 - Pachankis et al. (2020)	CBT: EQuIP (Empowering Queer Identities in Psychotherapy), a 10-session intervention adapted for sexual minority women from the ESTEEM protocol	Yes - adapted from the ESTEEM protocol, described above, with a focus on sexual minority women’s unique experiences	Center for Epidemiological Studies Depression Scale (CESD); Brief Symptom Inventory (BSI); Overall Depression Severity & Impairment Scale (ODSIS); Overall Anxiety Severity & Impairment Scale (OASIS); Sexual Minority Women's Rejection Sensitivity Scale (SMW-RSS); Sexual Orientation Concealment Scale (SOCS); Lesbian, Gay, and Bisexual Identity Scale - Internalized Homonegativity Subscale; Sexual Orientation Implicit Association Test; Difficulties of Emotion Regulation Scale - Short Form (DERSSF); Ruminative Responses Scale - Brooding Subscale (RRS); Simple RAS - Short Form (SRAS-SF)	As Pachankis et al. (2015); 60 (intent-to-treat)	1) Significant improvements in depressive symptoms (on CESD, ODSIS) and anxiety (OASIS) in immediate vs waitlist condition (large effect sizes), no significant condition - time interaction effects for minority stress processes or for universal processes (small effect sizes) 2) Stronger decreases in the proportion of immediate versus waitlist participants who continued to exceed the cut-off at three months (on ODSIS, not CESD, and on OASIS) 3) Significant improvements in all primary outcomes (large effect sizes), significant improvements in emotion regulation difficulties and rumination and marginally significant reductions in rejection sensitivity (small effect sizes for minority stress processes, small-medium effect sizes for universal processes) 4) Treatment effects generally continued to decrease at follow-up for mental and behavioural health outcomes, minority stress processes, and universal processes, BSI and rumination continuing to significantly decrease from post-treatment
5 - Maguen, Shipherd, & Harris (2005)	CBT: 12 weekly 60-minute sessions	Yes - session dedicated to hormone maintenance, surgeries, health care; session dedicated to disclosure, passing, socialisation; session dedicated to body issues and intimate relationships etc.	Beck Depression Inventory (BDI) State and Trait Anxiety Inventory (STAI) Network Orientation scale (NOS) - utilising social support networks in times of need Life Satisfaction Index (LSI)	N/A - individual scores; 6	Overall: - Anxiety and depression measures: Improvement - Social support: Increases in 4/6 participants - Life satisfaction indices: Decreased for the majority of participant, perhaps due to the multitude of life changes, including becoming unemployed and homeless
6 - Yadavaia & Hayes (2012)	ACT: 6-10 weekly 50-minute ACT sessions	Yes - explicitly addressing self-stigma around sexual orientation/internalised homophobia	Primary: - Daily Ratings of Thoughts About Sexual Orientation ((a) the degree to which negative thoughts about sexual orientation interfered in the participant's life, (b) the distress associated with those thoughts, (c) the believability of the thoughts, and (d) their frequency); Secondary: - Depression, Anxiety, and Stress Scales-21 (DASS-21); - Short Internalized Homonegativity Scale (SIHS); - Lesbian Internalized Homophobia Scale (LIHS); - WHOQOL-BREF (World Health Organization Quality of Life - Abbreviated Version); - AAQ-II (Acceptance and Action Questionnaire-II)	Hierarchical Linear Modeling (HLM); Mixed Model Repeated Measures; 5	Daily Ratings of Thoughts About Sexual Orientation: Improvements in interference and distress from baseline to the later time points in all participants; similar pattern for believability ratings; inconsistent and smaller changes for frequency ratings During baseline: No significant time effects for time for any of the rated dimensions During treatment: Frequency of thoughts did not change, but believability declined significantly, as did distress and self-reported interference IH: Improvement on SIHS and LIHS from pre-treatment by post-treatment (23%), by the 4-week follow-up (32%), and by the 12-week follow-up (40%) Depression, anxiety stress: No significant change on anxiety (from normal range at baseline); significant reduction in depression and stress (from moderate and mild range, respectively, at baseline) by follow-up; improvements in quality of life and psychological flexibility at 4-week follow-up
7 - Craig & Austin (2016)	CBT: AFFIRM intervention: eight module, manualised affirmative cognitive behavioural intervention	Yes - incorporating affirmative practices into traditional CBT models	Beck Depression Inventory (BDI-II); Stress Appraisal Measure for Adolescents (SAMA) - 3 subscales (challenge, threat, resources); Adolescent Proactive Coping Inventory (PCI-A) - Reflective Coping Subscale (RCS)	Repeated measures ANOVA - general linear model (GLM); T1-T2 = 30; T1-T3 = 17	Depression: Statistically significant reduction from T1 to T2, and from T1 to T3 Reflective coping: Non-significant increase from TI to T2; significant differences between T1 and T3 Stress appraisal: Threat appraisal: Significant decrease from T1 to T2, persisted to T3 Challenge appraisal: Significant increase from TI to T2, did not retain statistical significance to T3 Resource appraisal: Significant increase from T1 to T2, did not retain significance to T3
8 - Austin, Craig, & D’Souza (2018)	CBT: 2-day retreat - AFFIRM, described above	Yes - described above	Beck Depression Inventory (BDI-II); Adolescent Proactive Coping Inventory (PCI-A) - Reflective Coping Subscale (RCS)	Paired-sample t-tests (T1-T2, T1-T3, T2-T3); T1-T2 - 8, T1-T3, T2-T3 - 6	Depression: Statistically significant reduction from T1 to T2, from T1 to T3, nonsignificant reduction from T2 to T3; Mean scores at T2 and T3 remained in the BDI-II Severe range Coping: No significant differences from T1 to T2 or from T2 to T3
9 - Jabson Tree & Patterson (2019)	Online MBSR - 8 weeks, paralleled Kabat-Zinn's in-person MBSR	N/A	Perceived Stress Scale (PSS); Daily Experiences with Heteosexism Questionnaire (DEHQ)	1) Paired samples *t*-tests for changes in stress from baseline to postprogram and baseline to follow-up 2) Repeated-measures ANOVA tested mean values for each measure of stress against one another at the 3 time points; 17	Women: - Perceived stress (PSS): Significant decrease pre-post and pre-follow-up - Overall DEHQ and Vigilance subscale: Non-significant decrease pre-post, significant decrease pre-follow-up - Vicarious trauma subscale of the DEHQ: Significant decrease pre-post and pre-follow-up - Similar but less dramatic results on ITT analyses overall Men: - Perceived stress (PSS): Significant decrease pre-post, but not pre-follow-up, similar but less dramatic results on ITT analyses - DEHQ: No significant difference in either per-protocol or ITT analyses
10 - Cohen et al. (2021)	DBT; Other: Weekly 90-minute session over 10 consecutive weeks; participants were enrolled in individual psychotherapy and/or medication management concurrently	Yes - incorporates minority stress theory and adapts the teaching points of existing DBT skills to create Affirmative DBT Skills Training; including psychoeducation on the minority-specific psychological processes of rejection sensitivity, internalized stigma, and sexual orientation concealment	Difficulties of Emotion Regulation Scale (DERS); Overall Anxiety Severity & Impairment Scale (OASIS); Patient Health Questionnaire - Depression Module (PHQ-9); Gay-related Rejection Sensitivity Scale (GRS); Sexual Minority Women's Rejection Sensitivity Scale (SMW-RSS); Internalized Homophobia Scale (IHS); Sexual Orientation Concealment Scale (SOCS)	Clinically significant reliable change, with normative data used to calculate RCI acquired through the scales original articles; RCI not calculated for the GRS, SMW-RSS, IHS, and SOCS, as relevant data were not available; 6	Emotion regulation: Improvements in 5/6 participants (statistically significant for ~50% of the participants); Depressive symptoms: Improvements in 4/5 of the participants who reported a clinical level of depression at baseline (statistically significant for ~50% of the participants; Anxiety symptoms: Improvements in 3/4 of the participants who reported a clinical level of anxiety at baseline; GRS/SMW-RSS, IHS, and SOCS: Improvements in the majority of participants
11 - Hart et al. (2020)	CBT: Ten 1-hour, weekly sessions of CBT for treatment of social anxiety, related substance use in sexual situations, and HIV prevention	Yes - focus on participants' sexual and relationship history, goals for satisfying relationships and sex etc.	The Mini International Neuropsychiatric Interview version 6.0 (MINI 6.0); Anxiety Disorders Interview Schedule-IV-Lifetime (ADIS-IV), Social Phobia Section; Liebowitz Social Anxiety Scale (LSAS); The Social Interaction Anxiety Scale (SIAS) and Social Phobia Scale (SPS); Center for Epidemiologic Studies-Depression Scale (CESD); UCLA Loneliness Scale Version 3 (UCLA); Brief Fear of Negative Evaluation Scale, Straight-forward Items (BFNE-S)	Generalized estimating equations with robust estimators and unstructured correlation matrix addressing nonindependence of data across time points; Beta estimates for continuous measures and relative risk ratios (RR) for binary outcomes	Similar pattern of results using both intent-to-treat (*n* = 32) and completer (*n* = 21) samples; therefore, results of latter reported Social anxiety: - Significant reductions in the proportion of participants who met diagnostic criteria for social anxiety disorder from baseline to all timepoints - Significant reductions in mean scores on the LSAS, SIAS, SPS, BFNE-S between baseline and all time points Depression and loneliness: - Significant reduction in the proportion of patients with current major depressive episodes pre-post-treatment, non-significant differences for 3- and 6-month follow-up; - Significant reduction in mean scores on the CESD between baseline and all timepoints; - Significant reduction in mean scores on the UCLA between baseline and all timepoints
12 - Bluth et al. (2023)	Mindful Self-Compassion for Teens (MSC-T) - 8x1.5h sessions online, held over 8 days (1/day) for the first cohort, then 2x/week for 4 weeks for the second two cohorts	Yes - slight modifications to accommodate the needs of transgender adolescents e.g., ommission of body scan practice	Self-compassion scale: Youth (SCS-Y) Student life satisfaction scale (SLSS) Spielberger State Anxiety Scale - Short Form Patient Health Questionnaire-Depression Module (PHQ-9) Interpersonal needs questionnaire (INQ) Brief resilience scale (BRS)	One-way repeated measures ANOVAs; 26	Overall, main effect of time for all constructs across the study Depression: Significant decrease pre-post and pre-3-month follow-up Anxiety: Significant decrease pre-post (not observed at 3-month follow-up) Resilience: Significant increase pre-post (not observed at 3-month follow-up) Mindfulness: Significant increase pre-post and pre-3-month follow-up Self-compassion: Significant increase pre-post and pre-3-month follow-up
13 - Craig et al. (2021)	CBT: AFFIRM, described above - Online groups (eight weekly sessions) with 6-14 distinct participants in each age-appropriate (14-18, 19-24, 25+) group	Yes - AFFIRM, described above	Beck Depression Inventory (BDI-II); Brief COPE Inventory (BCI); Proactive Coping Inventory for Adolescents-A (PCI-A)-Reflective Coping Subscale (RCS); Stress Appraisal Measure for Adolescents (SAMA); Hope Scale (HS)	Linear multilevel models with restricted maximum likelihood estimation (REML) to test the effects of Time, Condition, and Time X Condition for all outcomes; age (centred at the mean of the whole sample = 22.34) included as a covariate in the model; Intervention (46), Control (50)	Compared to waitlist control, intervention condition participants experienced: - Significantly reduced depression - Significantly improved likelihood to appraise stress as challenge and to appraise that they had enough resources to deal with the stress - Significantly improved active coping, emotional support, positive framing, planning Marginally significant decrease in self-blame; no significant differences between the intervention and control conditions for substance use and behavioural disengagement - Increases for reflective coping or hope, but not statistically significant
14 - Pan et al. (2021)	CBT: ESTEEM, adapted for new contexts or populations	Yes - ESTEEM, described above, but with a different (Asian/Chinese) population	Chinese version of the PHQ-9 Chinese version of the GAD-7	Paired sample *t*-tests; 7	Reduction in the average score of depression and anxiety symptoms by approximately 7 and 5, respectively (medium-to-large improvement)
15 - Jackson et al. (2022)	CBT: Weekly 90-min group treatment sessions over 10 weeks	Yes - ESTEEM, described above, but adapted to recognise the intersectionality of racism and homophobia	Center for Epidemiological Studies Depression Scale (CESD); Overall Depression Severity & Impairment Scale (ODSIS); Overall Anxiety Severity & Impairment Scale (OASIS); Gay-related Rejection Sensitivity Scale (GRS); Self-Concealment Scale as previously modified for use with GBM; Internalized Homophobia Scale (IHS); Prolonged Activation and Anticipatory Race-Related Stress Scale - Psychological Subscale and Perseverative Cognitive Subscale; Racism-Related Vigilance Scale; Heterosexism in Racial Ethnic Minority Communities Subscale of the LGBT People of Color (POC) Microaggression Scale	*t*-tests - focusing on Hedge's *g* effect sizes 21 (baseline), 17 (3-month follow-up)	Depression symptoms and severity, anxiety, psychological distress, suicidal ideation: Decrease (very small effect sizes); Rejection sensitivity and concealment: Decrease (small effect sizes), but not internalised homophobia; Racial minority stress outcomes, including decreased anticipatory stress, race-related rumination, and race-related vigilance, and intersectional stress, including homophobia within one’s racial/ethnic community, racism within the LGBT community, and racism in dating and close relationships: Decrease (very small to small effect sizes)
16 - Poon et al. (2022)	DBT: 18-week comprehensive DBT-A (adaptation of DBT model for adolescents and their families) outpatient program offered to adolescents between the ages of 13-18, delivered with fidelity to the standard model, including a weekly multi family skills training group, individual therapy, 24/7 phone coaching, and a therapist consultation team	N/A	Difficulties of Emotion Regulation Scale (DERS); Beck Depression Inventory (BDI-II); Beck Anxiety Inventory; The dialectical behaviour therapy ways of coping checklist (DBT-WCCL); Boderline symptoms list (BSL)	1) Repeated-measures bootstrapped *t*-tests (two-tailed 0.05 p-values for treatment effects) - for LGBQ participants only 2) 2x2 mixed-model ANOVA to test group (LGBQ/non-LGBQ) effects on the outcomes; 16 - LGBQ for 1), 16 - LGBQ +23 - non-LGBQ for 2)	1) Significant improvements on all outcomes, apart from anxiety (mostly large effect sizes) 2) No significant group - time interaction effects on any of the outcomes (changes over time did not differ between LGBQ and non-LGBQ participants); statistically nonsignificant, but small to medium interaction effect sizes on the DERS, BDI-II, and WCCL-Skill Use (sexual minorities may benefit slightly more from DBT-A with respect to emotion regulation, depression, and effective skill use)

*Note.* All information was presented as found in the respective results sections of the primary reports; the same applies for evaluations of what is considered statistically or clinically significant, and effect sizes (although generally, statistically significant pertains to *p* < .05, clinically significant pertains to reductions in scores that either decrease to below clinical threshold of the respective scale or exceed the measurement error of the scales, and effect sizes are considered small (*d*/*g* = 0.2), medium (*d*/*g* = 0.5), and large (*d*/*g* ≥ 0.8) according to Cohen (1969), and the included studies seem to have adhered to this).

#### What Evidence-Based Cognitive and/or Behavioural Interventions for LGBTQ+ Populations Exist, and What, if Any, Specific Adaptations Do They Involve?

**CBT-based interventions:** Eleven studies involved CBT-based interventions, five of which featured the ESTEEM (Effective Skills to Empower Effective Men) intervention, or interventions based on it. ESTEEM was adapted via interviews with key stakeholders, including gay and bisexual men with depression and anxiety and expert providers, from Barlow et al.’s (2011) Unified Protocol to improve minority stress coping through emotion regulation, cognitive restructuring, and assertiveness training (identifying minority stress experiences; tracking cognitive, affective, and behavioural reactions to minority stress; attributing distress to minority stress rather than to personal failure; Pachankis, 2014; Pachankis et al., 2015).

Interventions based on ESTEEM included EQuiP (Empowering Queer Identities in Psychotherapy), which, following interviews with sexual minority women and expert clinicians, revised intervention contents to, for example, focus on sexual minority women’s unique experiences, including the intersection of sexism with other forms of oppression, exposure to sexual assault and harassment, or impact of gender norms (Pachankis et al., 2020).

ESTEEM was also adapted to more diverse contexts, populations, and ethnicities, with a view to address cultural contexts such as prioritisation of family needs and limited support from the health system (Pan et al., 2021), or to recognise intersectionality of racism and homophobia (Jackson et al., 2022). Adaptations occurred via key stakeholder feedback and by following the Assessment-Decision-Administration-Production-Topical Experts-Integration-Training-Testing (ADAPT-ITT) model (Wingood & DiClemente, 2008), a prescriptive method for adapting existing evidence-based interventions for new contexts or populations (Pan et al., 2021), as well as based on prior empirically supported group treatments for GBM of colour and guidance on psychotherapy for individuals who are both racial and sexual minorities (Jackson et al., 2022).

Three studies featured the AFFIRM intervention, a manualised affirmative cognitive behavioural intervention developed using case studies and community-based research, and participant feedback. AFFIRM targets young people with sexual and/or gender identity minority identities, focusing on improving coping and reducing depression. This occurs by explicitly acknowledging and validating the unique experiences of these populations, providing opportunities to understand and modify cognition (self-awareness, identifying risk, e.g., development of realistic alternative ways of thinking and behaving that affirm identities while integrating healthy ways of coping with internal/external stressors), mood (recognising the link between thoughts and feelings, e.g., how participants have learned to cope with identity-specific stressors), and behaviour (identifying strengths and ways of coping, e.g., connection to peer and adult allies) (Craig & Austin, 2016).

Furthermore, one study featured CBT for Trauma and Self-Care (CBT-TSC) including HIV counselling, another featured CBT for social anxiety including a focus on goals for satisfying relationships and sex, and a last study featured CBT with sessions dedicated to transgender-specific issues.

**DBT, mindfulness, ACT:** Two studies used DBT, one adapted by explicitly including minority stress psychoeducation; two studies featured mindfulness-based interventions (MBSR; Mindful Self-Compassion for Teens, MSC-T – with slight modifications to accommodate the needs of transgender adolescents). A final study featured ACT, explicitly addressing self-stigma around sexual orientation/internalised homophobia.

See Tudor-Sfetea and Topciu, 2024S, Appendix G for more details.

#### What Are the Outcomes of Evidence-Based Cognitive and/or Behavioural Interventions and Adaptations Targeting Mental Health in LGBTQ+ Populations?

##### Condition Differences Post-Intervention

Four studies, three of which were RCTs with inactive controls (#1, #3, #4) and one of which was a non-randomised experimental study (#13), all CBT-based, reported condition differences; #2, although an RCT, focused primarily on the effects of internalised homophobia.

###### Mental Health; Depression and Anxiety

Three studies (#1, #4, #13) reported significant improvements in depressive symptoms – although on different measures, and the two RCTs also at least marginally significant improvements in anxiety, all of which had medium-large effect sizes, maintained at follow-up where available. The fourth study (#3) focused on PTSD and showed significant improvements on all measures related to this, bar one subscale which showed a trend for significant difference; these effects were maintained or were trending towards this at follow-up.

###### Mental Health; Other Processes/Constructs and Minority Stress-Related Processes/Constructs

No significant differences were reported in any of the studies.

##### Pre-Post Differences

The remaining 11 studies reported pre-post intervention differences for variables of interest – however, three of the RCTs (#1, #2, #4) and the non-randomised experimental study (#13) also reported pre-post differences.

###### Mental Health; Depression and Anxiety

Fourteen studies reported results related to symptoms of depression, all of which showed improvements on at least one measure, ten of which (#1, #2, #4, #6, #7, #8, #11, #12, #13, #16) statistically or clinically significant, with medium-large effect sizes, generally maintained at follow-up. Ten studies reported results related to symptoms of anxiety, eight of which showed improvements, four of which (#1, #2, #4, #12) were at least marginally statistically or clinically significant, with medium-large effect sizes, of which two maintained the effects at follow-up. The study that focused on social anxiety (#11) showed significant improvements on all measures related to this.

###### Mental Health; Other Processes/Constructs

Two studies reported results related to emotion regulation, one of which (#16) showed significant improvements with large effect sizes, maintained at follow-up. Three studies reported results related to coping, only two of which (#7, #13) showed significant improvements between at least two timepoints, on different measures.

###### Minority Stress-Related Processes/Constructs

Six studies reported results related to internalised homophobia, three of which reported improvements on at least one analysis, one of which (#1) was statistically significant, with a large effect size, maintained at follow-up. Four studies reported results on rejection sensitivity, all of which showed improvements, but only one of which (#1) reported a significant result, with a large effect size. Finally, four studies reported results related to sexual orientation concealment, two of which reported improvements, none of which appeared to be significant, with small effect sizes.

### Risk of Bias in Studies

All four RCTs were judged to be at high risk of bias using the RoB 2 (Sterne et al., 2019), particularly due to high risk being identified in the measurement of outcome and missing data domains, respectively.

Of the 12 non-randomised studies, nine (#5, #7, #8, #9, #10, #11, #12, #14, #15) were judged to be at critical risk of bias using the ROBINS-I (Sterne et al., 2016), and three (#6, #13, #16) were judged to be at serious risk of bias. This was mostly due to critical scores in the confounding domain, as well as serious scores in the measurement of outcomes domain. Half of the studies also scored as serious on the selection of participants domain.

See Figures 2 and 3, generated using the Cochrane visualisation tool - robvis, McGuinness et al., 2021), and further details, in Tudor-Sfetea and Topciu, 2024S, Appendix H.

## Discussion

This review investigated evidence-based cognitive and/or behavioural interventions and adaptations for LGBTQ+ populations, complementing previous work (Bochicchio et al., 2022; Sheinfil et al., 2019; Van Der Pol-Harney & McAloon, 2019) by focusing specifically on cognitive and/or behavioural interventions and broadening the criteria to include participants of any age.

### Summary and Interpretation of Evidence

#### What Evidence-Based Cognitive and/or Behavioural Interventions for LGBTQ+ Populations Exist, and What, if any, Specific Adaptations Do They Involve?

The studies included in the review featured a range of therapeutic modalities (CBT – 11 studies; DBT – two studies; ACT – one study; mindfulness-based interventions – two studies). Of the CBT studies, eight involved versions of two protocolised interventions aimed specifically at LGBTQ+ individuals (ESTEEM, interventions based on it such as EQuiP, or adaptations to more diverse contexts or populations – five studies, and AFFIRM – three studies; see Table 3). Another four studies explicitly referred to LGBTQ+-specific adaptations, including a focus on stigma around sexual orientation, incorporating minority stress theory, or slight modifications to accommodate LGBTQ+ needs.

#### What Are the Outcomes of Evidence-Based Cognitive and/or Behavioural Interventions and Adaptations Targeting Mental Health in LGBTQ+ Populations?

When considering post-intervention differences between groups, of the four studies (three RCTs, one non-randomised experimental study) which reported this, three reported significant improvements in depressive symptoms, and the two RCTs also at least marginally significant improvements in anxiety. The fourth study, which focused on PTSD, showed significant improvements on most measures related to this. No significant differences were reported in terms of other mental health or minority stress-related processes/constructs.

When considering pre-post differences, these were reported in the remaining 11 studies as well as in three of the RCTs and the non-randomised experimental study. All the 14 studies investigating this showed improvement on at least one measure, ten being statistically/clinically significant. For anxiety, eight out of ten studies showed improvements, four thereof at least marginally statistically/clinically significant. The study that focused on social anxiety showed significant improvements on all measures related to this.

#### Reflections

Studies were heterogenous in terms of study designs, outcome measures, and analyses. Although the studies showed general improvements in certain areas such as depression, this is based on a variety of outcome measures (e.g., in some studies, significant improvements are seen on one outcome measure and not another, and viceversa – Pachankis et al., 2015, and Pachankis et al., 2020, respectively), as well as types of analysis (statistical significance, effect sizes, clinically significant reductions). This, together with the limitations of the studies (see below), raises questions about the strength and consistency of the evidence base.

The included studies also featured a heterogeneity of LGBTQ+ populations, such that the results cannot be generalised to any specific LGBTQ+ population without discussing the intersection of various identities (sexual, gender, racial, ethnic, social). Indeed, six studies focused on men, of which four included both gay and bisexual men, one included gay and bisexual men of colour, and one included sexual minority men in China. One study only focused specifically on women, three specifically on transgender individuals; moreover, most studies were conducted in North America. Therefore, findings may apply more to particular populations such as sexual minority men in North America, raising the question of whether other populations are the focus of enough relevant research.

Moreover, while a variety of transdiagnostic elements were featured in the studies’ interventions, mechanisms of change are not clearly differentiated such that the role of the minority stress-based adaptations remains largely unclear. Indeed, the most notable effects were observed for depression, while measures of minority stress (that is, proximal factors such as internalised homophobia, concealment, rejection sensitivity) showed less reliable improvements – or were not even explored at all (of the 16 included studies, only seven included such measures). Measures of other processes/constructs proposed to interact with minority stress (e.g., emotion regulation, unhelpful behaviours) were included in some studies, yet again, yielded unreliable results. While some authors (e.g., Pachankis et al., 2015) discuss that larger sample sizes would reveal such effects, it seems that certain components of non-empirically based treatment may also lead to improvements (Van Der Pol-Harney & McAloon, 2019).

Findings of this review were consistent with those of previous systematic reviews in that positive effects on mental health were reported, particularly in terms of symptoms of depression (Bochicchio et al., 2022; Sheinfil et al., 2019; Van Der Pol-Harney & McAloon, 2019), with comparable results for various modes of administration, including in-person, online, individual, or group (Bochicchio et al., 2022; Hobaica et al., 2018), and particularly for interventions based on CBT (Van Der Pol-Harney & McAloon, 2019). Furthermore, previous reviews also noted the paucity and heterogeneity of existing literature. However, while the cited reviews only explored interventions for young people, the current review expanded these to all ages, providing some evidence that results can be generalisable to adults as well, yet the intersection of these various characteristics and identities necessitates more in-depth exploration.

#### What Recommendations Could Be Made in Terms of Such Adaptations in Clinical Practice?

The heterogeneity in the studies leads to a limited ability to draw more precise conclusions about the effects of particular interventions for particular groups. Therefore, generic therapeutic competencies and metacompetencies (e.g., around engagement, therapeutic alliance and grasping clients’ ‘world views’, adapting interventions in response to client feedback, formulating and applying CBT models to the individual client etc., Roth & Pilling, 2007) may be especially important. Indeed, such competencies have been deemed important by some LGBTQ+ populations (McNamara & Wilson, 2020).

Applying these competencies to the needs of LGBTQ+ populations may also specifically mean adopting an affirmative approach, with clinicians being aware of LGBTQ+ issues (O’Shaughnessy & Speir, 2018), including minority stress, and receiving ongoing training on this (Boroughs et al., 2015; McNamara & Wilson, 2020). This may also mean adopting a more holistic approach, as LGBTQ+ individuals may benefit from addressing minority stress regardless of the format and drawing from social support to build resilience or reframe unhelpful beliefs (Alessi, 2014).

### Limitations of Evidence/Summary and Interpretation of Risk of Bias Evaluation

Searches yielded only 16 studies despite broad inclusion criteria. Only four studies used an RCT design, with the majority using a pre-post design with no control group, therefore not being able to establish a causal effect of the interventions. Moreover, sample sizes varied considerably, with some studies featuring very small sample sizes and some studies relying on the same sample, bringing into question statistical power and the relevance, reliability, and generalisability of results where statistical tests were not even used.

Risk of bias was evaluated as high in all four RCTs, and critical in nine of the non-randomised studies, with the remaining three non-randomised studies evaluated as serious. However, due to the nature of psychological interventions, domains regarding blinding participants and study personnel and measuring outcomes are intrinsically restricted. Nonetheless, almost all uncontrolled pre-post studies were evaluated as presenting critical risk of confounding, based on the ROBINS-I detailed guidance (Sterne et al., 2016), which recommends this where confounding is “inherently uncontrollable”. This may have led to a flooring effect.

### Limitations of the Review Process

Only English language and peer-reviewed studies were included, which limited the range of articles, potentially raising publication bias (Cuijpers et al., 2010). Our intention was to focus on the “gold standard” (peer-reviewed) literature as a first step, and research has found that “any unpublished studies identified in a given review may be an unrepresentative subset of all the unpublished studies in existence” (Higgins et al., 2023). A funnel plot was considered, but this was not possible, as treatment effects were not available for all included studies.

Additionally, we excluded certain populations (e.g., HIV-positive persons) and studies with outcomes related solely to drug use, and did not explicitly address outcomes related to suicidality or eating disorders. These areas were considered beyond the scope of this review due to the added complexity they would have brought. See Tudor-Sfetea and Topciu, 2024S, Appendix B for more details on these decisions.

Finally, while our search terms were developed in line with our inclusion/exclusion criteria, using the PICOS framework, and in collaboration with a University of Exeter librarian specialising in with Psychology, as well as via terms identified during the scoping search, we acknowledge that their use in their current form may have led to some potentially eligible studies not being retrieved. This is because terms such as “minority stress” encompass heterogenous sets of constructs which may have led to studies not being retrieved unless the constructs were explicitly part of the search string. This, of course, may in turn limit the representativeness of the studies and paint a relatively different picture of the landscape of the literature.

Our rationale for keeping terms rather broad was to keep a similar “detail level” of terms, one which was most likely to retrieve the most relevant results. Indeed, as discussed above, our findings are broadly in line with those of previous systematic reviews in the area, suggesting that the retrieved studies were mostly representative of the topic at hand. We provide a more extensive explanation in Tudor-Sfetea and Topciu, 2024S, Appendix B. The limited and heterogeneous nature of the evidence also restricted the possibility of exploring the data via meta-analyses and drawing more robust conclusions.

### Implications and Future Research Directions

To allow for more robust and more generalisable conclusions to be drawn, more consistency in outcome measures and general methodology is needed. This would allow for more meta-analyses to be conducted, and these should consider the impact of publication bias (Cuijpers et al., 2010). However, less strict methodologies may also offer pragmatic information on how interventions are administered and received in a variety of healthcare settings.

Moreover, as certain LGBTQ+ populations seem to be focused on more than others in the literature, more research needs to be carried out focusing on other LGBTQ+ populations, as well as discussing the intersection of various identities. More detailed investigations into specific mechanisms of change could also provide invaluable information as to the role of minority stress-based adaptations and what intervention aspects and therapeutic competencies are most important in producing positive outcomes, allowing for more investment and/or training in those areas.

### Conclusion

The review investigated evidence-based cognitive and/or behavioural interventions and adaptations for LGBTQ+ populations, revealing a range of therapeutic modalities and levels of adaptation. Findings showed largely positive effects, in line with previous systematic reviews – however, in the context of a paucity of the literature, with heterogeneity in terms of study designs, outcome measures, and analyses, as well as risk of bias evaluated as high or critical/serious (despite the possibility of a flooring effect). Limitations in terms of included studies and possible publication bias, as well as limited opportunity for generalisability and further exploration of the evidence to draw more robust conclusions are recognised. Suggestions for clinical practice are around the importance of generic therapeutic competencies and metacompetencies, and affirmative, potentially more holistic approaches. Suggestions for future research directions include more consistency in methodology, more focus on underserved LGBTQ+ populations and intersectionality, and more detailed investigations into mechanisms of change.

## Supplementary Materials

The Supplementary Materials contain the following items:

- **The preregistration** (Tudor-Sfetea & Topciu, 2022S)
- **Online Appendices**
  - *Appendix A* – Further explanation regarding deviations from the PROSPERO protocol (Tudor-Sfetea & Topciu, 2024S)
  - *Appendix B* – Further explanation regarding inclusion/exclusion criteria (Tudor-Sfetea & Topciu, 2024S)
  - *Appendix C* – Search terms and full search strategy/history (Tudor-Sfetea, 2023S)
  - *Appendix D* – Full extracted data table (Tudor-Sfetea, 2023S)
  - *Appendix E* – Overview of records excluded at full text review stage (Tudor-Sfetea & Topciu, 2024S)
  - *Appendix F* – Narrative summary of study and sample characteristics (Tudor-Sfetea & Topciu, 2024S)
  - *Appendix G* – Further details around study session numbers and duration (Tudor-Sfetea & Topciu, 2024S)
  - *Appendix H* – Further information regarding risk of bias in studies (Tudor-Sfetea & Topciu, 2024S)
  - *Appendix I* – Measures referenced in the data extraction table(s) (Tudor-Sfetea & Topciu, 2024S)

Tudor-SfeteaC.
 (2023S). A systematic review of evidence-based cognitive and/or behavioural interventions targeting mental health in LGBTQ+ populations
[Search terms, full search strategy/history, and full extracted data table]. PsychOpen. https://osf.io/zbu6r
10.32872/cpe.11323PMC1163674639678319

Tudor-SfeteaC.
TopciuR.
 (2022S). A systematic review of evidence-based cognitive and/or behavioural interventions targeting mental health in the LGBT+ community
[Preregistration]. PsychOpen. https://www.crd.york.ac.uk/prospero/display_record.php?ID=CRD42022243466
10.32872/cpe.11323PMC1163674639678319

Tudor-SfeteaC.
TopciuR.
 (2024S). Supplementary materials to "A systematic review of evidence-based cognitive and/or behavioural interventions targeting mental health in LGBTQ+ populations"
[Online appendices]. PsychOpen. 10.23668/psycharchives.15402
PMC1163674639678319

## Data Availability

For this article, a data set is freely available (Tudor-Sfetea, 2023S).

## Social Media Accounts

@cts504

## Reporting Guidelines

This systematic review was carried out in accordance with the updated PRISMA guidelines (Page et al., 2021).

## Preregistration

The work was registered on PROSPERO (International prospective register of systematic reviews) in April 2022 (CRD42022243466) (Tudor-Sfetea & Topciu, 2022S).
